# Characterization of Glutamate-Gated Chloride Channel in *Tribolium castaneum*

**DOI:** 10.3390/insects14070580

**Published:** 2023-06-25

**Authors:** Kun Qian, Chengyun Jiang, Daojie Guan, Anxiang Zhuang, Xiangkun Meng, Jianjun Wang

**Affiliations:** 1 College of Plant Protection, Yangzhou University, Yangzhou 225009, China; 2 College of Plant Protection, Jilin Agricultural University, Changchun 130118, China

**Keywords:** GluCl, exon 3, alternative splicing, *Tribolium castaneum*, variant

## Abstract

**Simple Summary:**

The glutamate-gated chloride channel (GluCl) is an important neurotransmitter receptor in the nervous system of invertebrates, and it is also a promising target for insecticide discovery. In the present study, three GluCl variants generated by alternative splicing of exon 3 were cloned from the red flour beetle *Tribolium castaneum*, and their sequence features, genomic structures, and expression profiles as well as the homology modeling of TcGluCl were studied. These results provide valuable information for studying the specific function of the insect GluCl variant.

**Abstract:**

The glutamate-gated chloride channels (GluCls) play essential roles in signal transduction by regulating fast inhibitory synaptic transmission in the nervous system of invertebrates. While there is only one GluCl subunit in the insect, the diversity of insect GluCls is broadened by alternative splicing. In the present study, three *TcGluCl* variant genes were cloned from the red flour beetle *Tribolium castaneum*. Analysis of the characteristics of TcGluCls including sequence features, genomic structures, and alternative splicing revealed that TcGluCls had the typical structural features of GluCls and showed high homologies with the GluCls from other insect orders. The TcGluCl-encoding gene consists of nine exons and three variants (*TcGluCl-3a*, *TcGluCl-3b*, and *TcGluCl-3c*) were generated by the alternative splicing of exon 3, which was a highly conserved alternative splicing site in insect GluCls. Homology modeling of TcGluCl-3a showed that the exon 3 coding protein located at the N-terminal extracellular domain, and there were no steric clashes encountered between the exon 3 coding region and ivermectin/glutamate binding pocket, which indicated that the alternative splicing of exon 3 might have no impact on the binding of GluCls to glutamate and insecticide. In addition to the head tissue, *TcGluCl-3a* and *TcGluCl-3c* also had high expressions in the ovary and testis of *T. castaneum*, whereas *TcGluCl-3b* showed high expression in the midgut, suggesting the diverse physiological functions of TcGluCl variants in *T. castaneum*. The total *TcGluCl* and three variants showed the highest expression levels in the early stage larvae. The expressions of *TcGluCl*, *TcGluCl-3b*, and *TcGluCl-3c* were significantly increased from the late-stage larvae to the early stage pupae and indicated that the *TcGluCl* might be involved in the growth and development of *T. castaneum*. These results are helpful to further understand the molecular characteristics of insect GluCls and provide foundations for studying the specific function of the GluCl variant.

## 1. Introduction

The glutamate-gated chloride channels (GluCls) belong to the cys-loop ligand-gated ion channels (LGICs) superfamily and play an important role in signal transduction by mediating fast inhibitory synaptic transmission in the nervous system [1]. GluCls have only been found in invertebrates to date, and they are proposed to be an ideal target protein for insecticide action in pest management [2]. However, only a few insecticides targeting GluCls are developed including the macrocyclic lactone abamectin, ivermectin, and the phenylpyrazole fipronil [3,4]. GluCls are formed by five subunits, and each subunit has an N-terminal extracellular domain and four transmembrane helices, which play essential roles in ligand binding and channel formation, respectively [5]. The GluCl-encoding gene was firstly cloned from nematode *Caenorhabditis elegans* and six GluCl subunit genes including four *α* subunits and one *β* subunit were found in *C. elegans* [6,7]. Six GluCl subunit genes (*GluCl1-6*), which all display high similarities with the nematode GluCl *α* subunit orthologue, were identified from the genome of mite *Tetranychus urticae* [8]. Whereas only one orthologous gene encoding the α subunit was found in different insect species such as *Drosophila melanogaster*, *Bombyx mori*, *Locusta migratoria*, and *Apis mellifera* [9,10,11,12]. Although a single GluCl gene exists in insects, however, studies revealed that diverse pharmacological properties of GluCls were found in insects which might be formed by different splice variants of GluCl [11,13,14].

Alternative splicing results in transcriptome diversification and a large portion of protein-encoding genes in multicellular organisms are alternatively spliced [15]. Alternative splicing of exon 3 of the *GluCl* gene widely exists in insects and three variants namely *GluCl-3a*, *GluCl-3b*, and *GluCl-3c* are generated in general [2]. Expression profile studies showed that the three variants had different distributions in insects. In addition to the head tissue, GluCl variants also had high expression levels in the leg, gut, cuticle, and malpighian tubules of insects [2,16,17]. Electrophysiological studies also revealed that the functional GluCls formed by different variants showed diverse pharmacological properties to ligands [10,16]. These results indicate that the variants derive from alternative splicing of GluCl exon 3 may have different physiological functions in insect, while the specific role of each variant is largely unclear yet.

The red flour beetle *Tribolium castaneum* (Coleoptera) is a major pest of stored agricultural products such as flour, wheat, corn, and beans, and it is also an important model insect for studying the functions of various genes. Previous analysis of *T. castaneum* genome found that three variants (*TcGluCl-3a*, *TcGluCl-3b*, and *TcGluCl-3c*) were generated by alternative splicing of GluCl exon 3 in beetles [18]. In this study, the three TcGluCl variant genes were cloned from *T. castaneum*, and their characteristics, genomic structures, alternative splicing, and spatial expressions were analyzed. More specifically, the effects of alternative splicing of exon 3 on the binding of TcGluCl to ligands were studied using homology modeling.

## 2. Materials and Methods

### 2.1. Insects

The Georgia-1 (GA-1) strain of *T. castaneum* was used in this study. The whole life cycle of *T. castaneum* was reared on 5% (*w*/*w*) yeasted flour under the conditions of 30 °C and 40% relative humidity.

### 2.2. Total RNA Isolation and Reverse Transcription

Total RNA was isolated from 15-day-old larvae using TaKaRa MiniBEST universal RNA Extraction Kit (TaKaRa, Dalian, China) following the manufacturer’s instructions. The first strand cDNA was synthesized from 1 μg of total RNA employing the PrimeScript 1st Strand cDNA Synthesis Kit (TaKaRa, Dalian, China) according to the manufacturer’s instructions.

### 2.3. Cloning and Sequence Analysis of TcGluCl

The specific primer pairs used for *TcGluCl* cloning were designed based on the genome (project accession number PRJNA12540) sequence of *TcGluCl* (Table S1). Polymerase chain reaction (PCR) containing cDNA template, primers, and DNA polymerase was used to amplify the open reading frame of *TcGluCl*. The PCR reaction was performed with a condition of 95 °C for 30 s, 10 cycles at 95 °C for 30 s, 55–50 °C (decreasing by −0.5 °C/cycle) for 30 s, and 72 °C for 2 min, and followed by 20 cycles of 95 °C for 30 s, 50 °C for 30 s, and 72 °C for 2 min, then a 72 °C for 5 min extension at the end. The product generated from PCR was purified and sequenced, and the TcGluCl variants were identified based on random nucleotide sequencing.

Amino acid sequence derived from the cloned *TcGluCl* was aligned with GluCls from other insects including *D. melanogaster* (GenBank accession number AAG40735), *B. mori* (GenBank accession number NP_001275941), *Laodelphax striatellus* (GenBank accession number ANN12556) and *A. mellifera* (GenBank accession number DQ667185) using CLUSTALW [19]. The conserved motifs of insect GluCls such as six ligand binding loops (Loop A–F) and four transmembrane domains (TM1–4) were predicted using the ExPASy ScanProsite (http://prosite.expasy.org/scanprosite/, accessed on 10 August 2022) and TMHMM 2.0 (http://www.cbs.dtu.dk/services/TMHMM-2.0/, accessed on 10 August 2022), respectively, or were identified based on previous report [2]. The signal peptide in insect GluCl was predicted using SignalP 4.1 server (http://www.cbs.dtu.dk/services/SignalP/, accessed on 10 August 2022). Molecular weight and theoretical isoelectric point (pI) of the deduced TcGluCl protein sequences were predicted employing the Compute pI/Mw tool (https://web.expasy.org/protparam/, accessed on 10 August 2022). The genomic and cDNA sequences of *TcGluCl* were manually aligned to locate the exon–intron boundaries. Genomic structures of TcGluCl variants were constructed employing the IBS 1.0 software [20]. Amino acid sequences encoded by GluCl exon 3 from different insects were aligned and compared and were also used in phylogenetic tree construction using neighbor-joining method in MEGA 7 with a bootstrapping of 1000 iterations.

### 2.4. Homology Modelling of TcGluCl

A homology modeling of TcGluCl-3a was created using Swiss Pdb-Viewer program via the Expasy web server (https://swissmodel.expasy.org/, accessed on 1 March 2023) [21]. The crystal structure of GluCl α subunit from *Caenorhabditis elegans* (3RIF.pdb) was used as comparative model for creating TcGluCl-3a structure [22]. To identify the potential binding site of ligand, the bound glutamate and ivermectin molecules were added to the homology modeling through superposition with 3RIF.pdb coordinates. The position of exon 3 coding protein was indicated through amino acid alignment.

### 2.5. Reverse Transcription Quantitative PCR (RT-qPCR)

Several specific tissue samples including head, midgut, malpighian tubules, testis, ovary, cuticle, and fat body were dissected from the 7-day-old male or female adults. Six whole bodies in different developmental stages of larvae (1-day-old, 10-day-old and 20-day-old), pupae (1-day-old and 5-day-old), male adults (1-day-old and 7-day-old) and female adults (1-day-old and 7-day-old) were collected as a sample. Each sample was replicated three times. Total RNAs of different tissues were isolated using the method described above. The cDNA templates used for gene expression determination were synthesized using PrimeScript RT reagent Kit with gDNA Eraser (TaKaRa, Dalian, China) following the manufacturer’s instructions. Specific primers of each TcGluCl variant were designed with a primer anchored to exon 3 and the counterpart primer located on either side of exon 3. Gene expression levels were determined by RT-qPCR reactions using TB Green Premix Ex Taq (TaKaRa, Dalian, China) with gene-specific primers (Table S1) and the stably expressed reference gene encoding ribosomal protein S3 (rps3, GenBank accession number CB335975). The reaction volume containing 10 μL TB Premix EX Taq™ II (2×), 6.4 μL ultrapure water, 2 μL cDNA template, and 0.8 μL (10 μM) of each primer. RT-qPCR reaction was performed on a CFX96 Real-Time PCR Detection System (Bio-Rad Laboratories, Inc., Hercules, CA, USA) with a condition of 95 °C for 30 s, 40 cycles of 95 °C for 5 s, and 60 °C for 30 s. Relative gene expressions were normalized to the reference gene *rps3* in the same sample and calculated using 2^−ΔΔCT^ method [23].

### 2.6. Statistical Analysis

Gene expression determination experiments were performed in three independent biological replicates. Data were presented as mean values ± standard error of the mean (SEM). One-way ANOVA with post hoc Tukey’s HSD test was used for multiple comparisons of parametric data. All statistical analyses were performed using SPSS software (SPSS 13.0 for Windows; SPSS Inc., Chicago, IL, USA).

## 3. Results

### 3.1. Cloning and Structural Characteristics of TcGluCl

**Figure 1:**
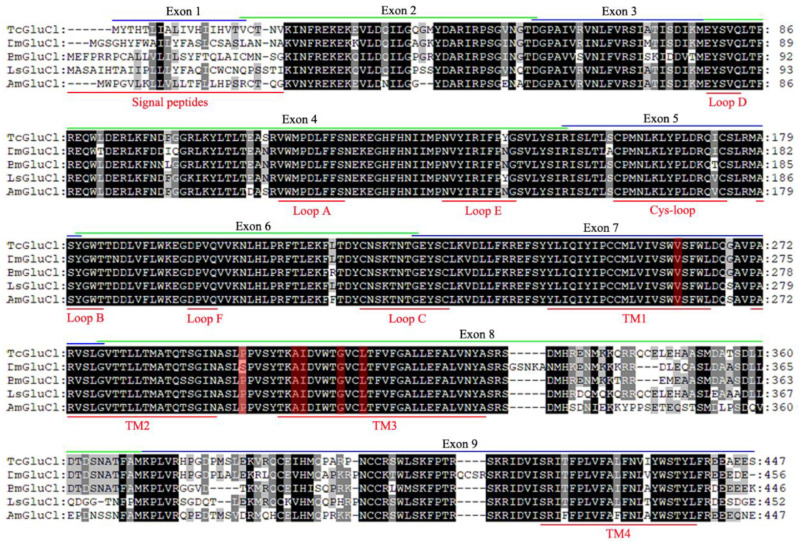
Amino acid sequence alignments of GluCls from *T. castaneum*, *D. melanogaster*, *B. mori*, *L. striatellus*, and *A. mellifera*. The signal peptide, six ligand binding loops (Loop A–F), and four conserved transmembrane regions (TM1–4) of GluCls were marked and underlined in red. The nine exon coding sequences were lined in green or blue and marked above the alignments. The red background amino acids indicate the mutations associated with insecticide resistance in insects and mites. In the alignments, amino acids with identities of 100%, more than 80%, or less than 60% were indicated with black, grey and white background, respectively.

The full length of *TcGluCl* was cloned from *T. castaneum* and three variant genes (*TcGluCl-3a*, *TcGluCl-3b*, and *TcGluCl-3c*) were identified based on nucleotide sequencing. The nucleotide sequences of *TcGluCl-3a*, *TcGluCl-3b*, and *TcGluCl-3c* were deposited to the NCBI Genbank with accession numbers OR148383, OR148384, and OR148385, respectively. All of the three *TcGluCl* variant genes contain an open reading frame of 1344 bp encoding 447 amino acids with a predicted molecular weight of about 51.6 kDa and pI of about 8.2. *TcGluCl-3a*, *TcGluCl-3b*, and *TcGluCl-3c* shared the same nucleotide sequences except for the nucleotides located at 168–231 bp. Alignments of TcGluCl-3a and GluCls from other representative insects revealed that insect GluCls had high sequence homologies with each other (Figure 1). TcGluCl-3a showed amino acid identities of 83.3%, 82.4%, 82.8%, and 84.0% with the GluCls of *D. melanogaster*, *A. mellifera*, *B. mori*, and *L. striatellus*, respectively. Particularly, the important regions of GluCls including the six ligand binding loops (Loop A–F) and four transmembrane domains (TM1–4), which play crucial roles in ligand binding and channel formation, respectively, were highly conserved in insect GluCls (Figure 1). Meanwhile, the main sequence differences of insect GluCls were located in the signal peptide of the N-terminal extracellular domain and the intracellular loop between TM3 and TM4 (Figure 1).

### 3.2. Genomic Structures of Three TcGluCl Variants

**Figure 2:**
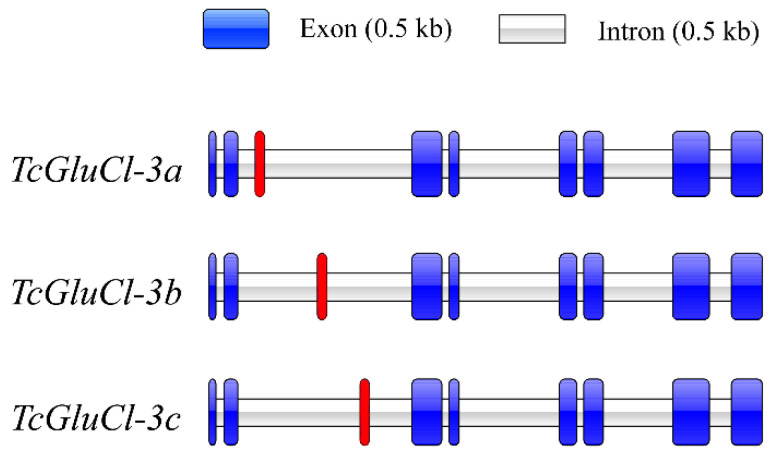
Analysis of the genome structures of three TcGluCl variants. The red background exon indicates exon 3.

The cloned mRNA sequences of three *TcGluCls* were mapped to the genomic sequence of *TcGluCl*, respectively, and results revealed that the genomic sequence of *TcGluCl* consists of nine exons and eight introns (Table S2). Analysis of genomic structures of the three *TcGluCls* showed that three *TcGluCls* shared the same exon 1–2 and exon 4–9, but had a different exon 3 due to the alternative splicing (Figure 2, Table S2).

### 3.3. Alternative Splicing of Exon 3 of TcGluCl

**Figure 3:**
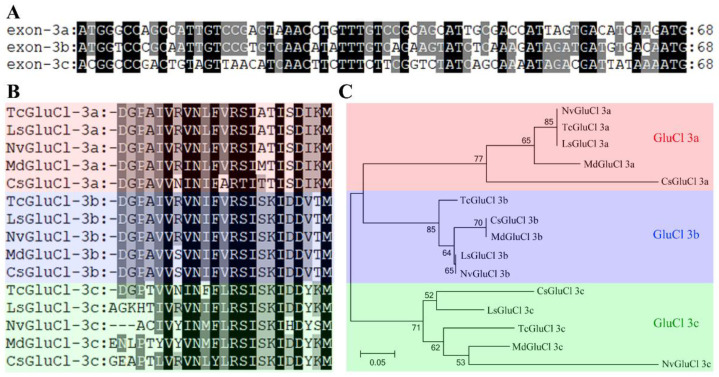
Sequence alignments and phylogenetic analysis of TcGluCl variants. (**A**) Nucleotide sequence alignments of exon 3 of three TcGluCl variants. The identical bases were indicated with black or grey background (**B**) Amino acid sequence alignments of exon 3 variants from *T. castaneum*, *L. striatellus*, *N. vitripennis*, *M. domestica*, and *C. suppressalis*. Amino acids with identities of 100% or more than 80% were indicated with black and grey background, respectively. (**C**) Phylogenetic analysis of GluCls variants from *T. castaneum*, *L. striatellus*, *N. vitripennis*, *M. domestica*, and *C. suppressalis*. The exon 3 coding amino acid sequences were used in phylogenetic tree construction. The clusters of insect GluCl 3a, GluCl 3b and GluCl 3c were marked with red, blue and green background, respectively.

The exon 3 of *TcGluCl* had a length of 68 bp encodes 23 amino acids. Alternative splicing of exon 3 generated three *TcGluCl* variants, namely *TcGluCl-3a*, *TcGluCl-3b*, and *TcGluCl-3c*. The three alternative exons of *TcGluCl* (exon 3a, exon 3b, and exon 3c) shared nucleotide and amino acid identities of 47.1–66.2% and 56.5–73.9%, respectively, and they also showed high identities to their corresponding variants in different insect orders such as *L. striatellus* (Hemipteran), *Nasonia vitripennis* (Hymenopteran), *Musca domestica* (Dipteran), and *Chilo suppressalis* (Lepidopteran) (Figure 3A,B). Specifically, exon 3a and exon 3b were highly conserved among insect GluCls with amino acid identities of 69.6–100% and 91.3–100%, respectively, while the exon3c showed relatively low conservation among insect GluCls with amino acid identities of 50.0–70.8% (Figure 3B). Phylogenetic analysis of GluCl exon 3 revealed that the alternative exons showed close relationships with their corresponding homologs in insects (Figure 3C).

### 3.4. Molecular Modeling of TcGluCl

**Figure 4:**
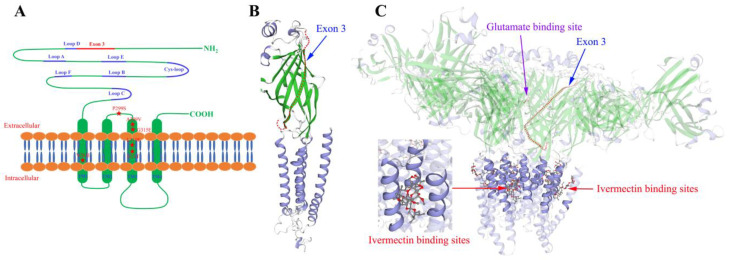
Structure diagrams of TcGluCl. (**A**) Planar structure of a single TcGluCl subunit. The positions of exon 3 and six ligand binding loops were marked, and the amino acid mutations associated with insecticide resistance in insects and mites were indicated using five-pointed star. (**B**) Molecular modeling of a single TcGluCl-3a subunit. The red dotted line indicates the exon 3 coding region. (**C**) Homology modeling of pentameric TcGluCl formed by TcGluCl-3a. The red and purple arrows indicate the ivermectin and glutamate binding sites, respectively. The red dotted line represents the exon 3 coding region.

The exon 3 coding region adjacently lies upstream of Loop D, which might involve in the ligand binding of TcGluCl (Figure 1 and Figure 4A). In order to explore the spatial relationship between the exon 3 coding protein and the ligand binding site of TcGluCl, a homology modeling of TcGluCl-3a was generated employing the crystal structure of *C. elegans* GluCl orthologue in a complex with ivermectin as a homology template (3RIF.pdb). A single subunit modeling of TcGluCl-3a showed that the exon 3 coding protein was located in the N-terminal extracellular domain of GluCl (Figure 4B). A pentamer modeling of TcGluCl-3a showed that the insecticide ivermectin binding pocket located among the transmembrane helices of TM1, TM2, and TM3, where most of the reported mutations associated with insecticide resistance located, and there were no steric clashes encountered between the exon 3 coding region and ivermectin binding site (Figure 4C). The exon 3 coding region lies close to the glutamate binding pocket, however, there was no direct contact between them (Figure 4C).

### 3.5. Spatial and Temporal Expressions of TcGluCls in T. castaneum

**Figure 5:**
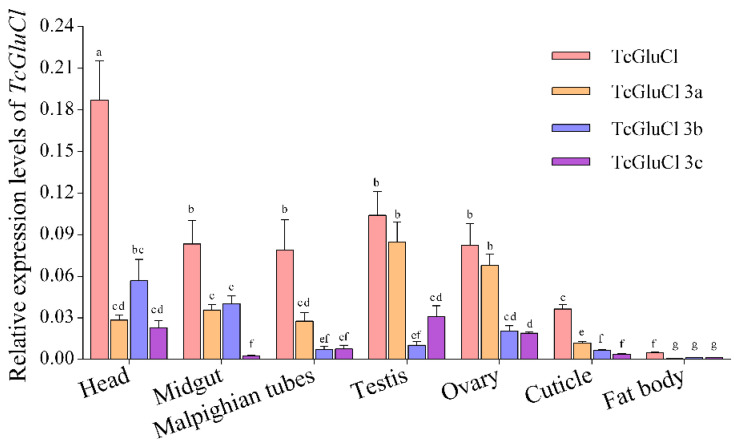
Spatial expressions of total *TcGluCl* and three variants in seven specific tissues of *T. castaneum*. Data are expressed as mean ± SEM (*n* = 3 biologically independent replicates). Histogram bars annotated with the same lowercase letters are not significantly different (one-way ANOVA, *p* < 0.05).

The transcriptional levels of *TcGluCl-3a*, *TcGluCl-3b*, and *TcGluCl-3c* as well as the total *TcGluCl* (including the three variants) in several specific tissues of *T. castaneum* were determined. Results found that the three variants were expressed in all of the tested tissues with different expression levels (Figure 5). The total *TcGluCl* had the highest expression level in head tissue, furthermore, *TcGluCl* also showed high expression levels in the midgut, malpighian tubules, testis, ovary, and cuticle of *T. castaneum*. *TcGluCl-3a* had relatively high expressions in the ovary and testis tissues associated with reproduction, and *TcGluCl-3c* showed high expressions in the head, ovary, and testis, whereas *TcGluCl-3b* was abundantly expressed in the head and midgut (Figure 5).

**Figure 6:**
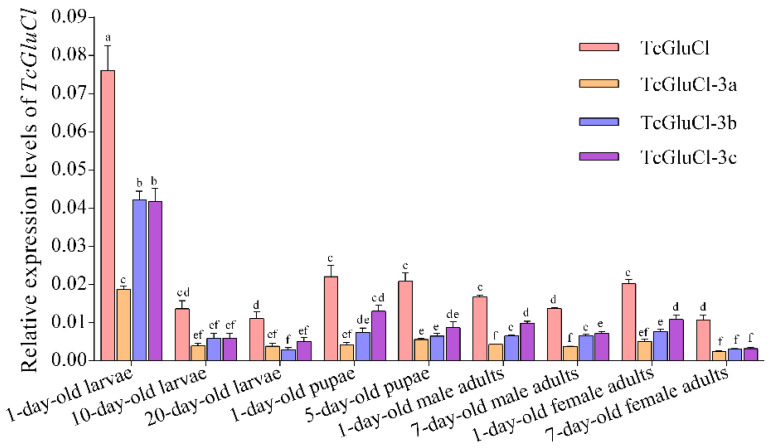
Temporal expressions of total *TcGluCl* and three variants in the larvae (1-day-old, 10-day-old, and 20-day-old), pupae (1-day-old and 5-day-old), and adults (1-day-old and 7-day-old) of *T. castaneum*. Data are expressed as mean ± SEM (*n* = 3 biologically independent replicates). Histogram bars annotated with the same lowercase letters are not significantly different (one-way ANOVA, *p* < 0.05).

The transcriptional levels of the total *TcGluCl* and three variants in several different developmental stages of *T. castaneum* were determined. The results showed that *TcGluCl-3a*, *TcGluCl-3b*, and *TcGluCl-3c*, as well as the total TcGluCl, had the highest expression levels in the early stage larvae and their expressions were dramatically decreased during larvae development from 1-day-old larvae to 10-day-old larvae, whereas there were no significant differences of expressions of *TcGluCl* and three variants in 10-day-old larvae and 20-day-old larvae (Figure 6). The expressions of *TcGluCl*, *TcGluCl-3b*, and *TcGluCl-3c* were significantly increased from the late-stage larvae (20-day-old larvae) to the early stage pupae (1-day-old pupae), while the expressions of *TcGluCl* and three variants were not significantly changed in the pupae stages and early stage adults (1-day-old adults). Furthermore, the expressions of total *TcGluCl* and *TcGluCl-3c* were decreased during adult development from 1-day-old adult to 7-day-old adult (Figure 6).

## 4. Discussion

The GluCl regulates fast inhibitory synaptic transmission in the nervous system of invertebrates, and it is a promising target for insecticide discovery [1]. *GluCl* genes have been widely cloned from various insects, while their characteristics and physiological functions were not fully understood. In the present study, sequence analysis showed that TcGluCl were highly conserved with GluCls from other insect orders including hemipteran, hymenopteran, dipteran, and lepidopteran with amino acid identities of more than 82.0%, especially in the important ligand binding domains and transmembrane regions. The high identities of insect GluCls might be partly responsible for the broad-spectrum insecticidal activities of abamectin and fipronil.

Generally, the open reading frame of insect *GluCl* consists of 9–11 exons, such as 10 exons in the *GluCl* of *Plutella xylostella* and *B. mori* [10,24], 10–11 exons in the *GluCl* of *Anopheles gambiae* and nine exons in *TcGluCl* in this study [25]. Although the numbers of exons were different, however, the alternative splicing site of insect *GluCl* was conserved. Alternative splicing of exon 3 widely happened in insect *GluCl* and three alternative exons (exon 3a, exon 3b, and exon 3c) were generated [2,10,12,16,17,25]. The three alternative exons 3 encoded 22–24 amino acid residues and showed very high sequence identities with their corresponding variants in different insects. In addition to the alternative splicing of exon 3, GluCl variants generated by the alternative splicing of exon 9 were found in *C. suppressalis*, *B. mori*, *P. xylostella*, *L. striatellus*, and *D. melanogaster* [2,10,17,26,27]. However, we did not identify the variant derived from alternative splicing of exon 9 in *T. castaneum*. The high homology and conserved alternative splicing site of GluCl suggested that insect *GluCl* was a highly conserved gene during biological evolution.

Even though there is only one GluCl subunit in insects, the diversity of insect GluCl is broadened by alternative splicing which may lead to diverse pharmacological properties of GluCls. At least two types of GluCls with different responses to agonists or channel blockers were found in Periplaneta americana, Locusta migratoria, and *A. mellifera* [11,13,14]. Electrophysiological studies of GluCl variants from housefly *Musca domestica* revealed that the three variants (*MdGluClA*, *MdGluClB*, and *MdGluClC*) generated by alternative splicing of exon 3 showed similar sensitivities (the 50% effective concentration, EC_50_, and maximum current amplitude, *I*_max_) to agonist glutamate and the allosteric activator ivermectin when they were singly or co-expressed in *Xenopus* oocytes, whereas showed diverse sensitivities (the 50% inhibitory concentration, IC_50_) to the channel blockers fipronil and picrotoxinin [16]. In the studies of GluCl variants from *B. mori*, the three functional GluCls formed by single variant GluCl-3a, GluCl-3b, or GluCl-3c had similar EC_50_ values for glutamate and ivermectin, however, the *I*_max_ values induced by glutamate and ivermectin differed significantly between variants [10]. In the present study, homology modeling of TcGluCl showed that the exon 3 coding protein was located at the N-terminal extracellular domain, and there were no steric clashes encountered between the exon 3 coding region and ivermectin/glutamate binding pocket. Similar results were also found in the GluCl variant from *Anopheles gambiae* [25]. These findings indicated that functionally indistinguishable, but pharmacologically distinct GluCls could be formed in insects, and the alternative splicing of exon 3 might has no impact on the binding of GluCls to glutamate and insecticide.

As a major neurotransmitter receptor in the nervous system, GluCl variants were predominantly expressed in the head or brain of insects [2,16,17]. Precise immunolocalization of the brain from *A. mellifera* found that the exon 3a variant was mainly located in the neuropils of the brain, whereas the exon 3b variant was mostly expressed in cell bodies [28]. The different expression distributions of variants in the brain might suggest the diverse physiological functions of GluCl variants in *A. mellifera*. In addition to the head tissue, the GluCl variants were also found to be highly expressed in the leg, gut, cuticle, and ovary tissues of insects [2,16,17]. For example, *LsGluCl-3a* of *L. striatellus* and *MdGluCl-3c* of *M. domestica* were abundantly expressed in the leg tissue, *CsGluCl-3a* of *C. suppressalis* was mainly expressed in the brain and nerve cord and had very low expression levels in other tissues, whereas *CsGluCl-3b* showed high expression in the cuticle and *CsGluCl-3c* had high expressions in the cuticle, ovary, and gut [2,16,17]. Theoretically, the expression profile of a gene was related to its physiological role in an organism. In addition to the neurotransmitter transmission function, insect GluCls were found to be associated with the biosynthesis of juvenile hormone in *Diploptera punctate* [29,30], the olfactory learning and memory of *A. mellifera* [12,31,32], the inhibitory action on the olfactory system and rhythmic light avoidance of *D. melanogaster* [33,34], and the flight and waking control of *L. migratoria* [11]. Temporal expressions of TcGluCls revealed that the total *TcGluCl* and three variants had the highest expression levels in the early stage larvae, and the expressions of *TcGluCl*, *TcGluCl-3b*, and *TcGluCl-3c* were significantly increased from the late-stage larvae to the early stage pupae. Similar results were also found in *C. suppressalis* [2] and indicated that the *GluCl* might involve in the growth and development of insects. Spatial expressions of the TcGluCl variants showed that the TcGluCls were abundantly expressed in the head, midgut, malpighian tubules, testis, ovary, and cuticle tissues. Specifically, *TcGluCl-3a* and *TcGluCl-3c* had high expressions in the ovary and testis tissues, and *TcGluCl-3b* showed high expression in the head and midgut. The high expressions of *TcGluCl-3a* and *TcGluCl-3c* in ovary and testis tissues might suggest their roles involved in the development and reproduction of *T. castaneum*. Since studies have found that the GluCl gene *avr-14* was implicated in the gamete production and embryogenesis of the filarial worm *Brugia malayi* [35], and knockdown of *GluCl* resulted in the decreased larval weight and pupation rate of *C. suppressalis* and reduced the hatch rate of *Helicoverpa zea* [2,36]. However, the specific roles of GluCl variants in the leg, gut, and reproductive tissues of insects remain unclear and need further study.

## 5. Conclusions

In summary, three TcGluCl variant genes generated by alternative splicing of exon 3 were cloned from *T. castaneum*, and their characteristics, genomic structures, expression profiles as well as the effect of alternative splicing of exon 3 on the binding of TcGluCl to ligands were analyzed. Our results are helpful to further understand the molecular characteristics of insect GluCls and provide foundations for studying the specific function of GluCl variants.

## Data Availability

The data presented in this study are available from the corresponding author upon reasonable request.

## Supplementary Materials

The following supporting information can be downloaded at: https://www.mdpi.com/article/10.3390/insects14070580/s1. Table S1. The primers used in this study. Table S2. The lengths of exons and introns in TcGluCl genomic sequence.
