# Supplementary material for: Characterization of Glutamate-Gated Chloride Channel in Tribolium castaneum
Source: Insects. 2023 Jun 25;14(7):580. doi: 10.3390/insects14070580 (PMC10380907; doi:10.3390/insects14070580)
Supplement: Supplementary file 1 [file insects-14-00580-s001.zip › insects-2431355-supplementary.pdf]

Table S1. The primers used in this study

Description	Primer name	Sequence (5′ to 3′)
ORF amplification	TcGluCl-F1	AATCGGTGAGGTTGAGTCTTGG
	TcGluCl-R1	TACTATTACGGTCGCCGCAAGG
RT-qPCR	TcGluCl-F2	CCACAACATTATTATGCCCAACG
	TcGluCl-R2	GGGTAGAGCTTGAGGTTCATCG
	TcGluCl 3a-F	CAGCCATTGTCCGAGTAAACCT
	TcGluCl 3a-R	TTGAGTCTTTCGTCTAGCCATT
	TcGluCl 3b-F	GTCCCGCAATTGTCCGTGTCAAC
	TcGluCl 3b-R	CGACCTCCAAAGTCGTTAAACTT
	TcGluCl 3c-F	GCCCGACTGTAGTTAACATCAAC
	TcGluCl 3c-R	CGACTGGCTTCAGTTAAAGTAAG
	rps3-F	ACCGTCGTATTCGTGAATTGAC
	rps3-R	ACCTCGATACACCATAGCAAGC

Table S2. The lengths of exons and introns in *TcGluCl* genomic sequence

Exon	Length (bp)	Intron	Length (bp)
exon 1	53	intron 1	58
exon 2	113	intron 2a	129
exon 3a	68	intron 3a	1120
exon 3b	68	intron 2b	602
exon 3c	68	intron 3b	647
exon 4	224	intron 2c	927
exon 5	83	intron 3c	322
exon 6	135	intron 4	54
exon 7	153	intron 5	754
exon 8	281	intron 6	52
exon 9	234	intron 7	525
		intron 8	163
